# Definition of a microbial signature as a predictor of endoscopic post-surgical recurrence in patients with Crohn’s disease

**DOI:** 10.3389/fmmed.2023.1046414

**Published:** 2023-02-03

**Authors:** Lia Oliver, Blau Camps, David Julià-Bergkvist, Joan Amoedo, Sara Ramió-Pujol, Marta Malagón, Anna Bahí, Paola Torres, Eugeni Domènech, Jordi Guardiola, Mariona Serra-Pagès, Jesus Garcia-Gil, Xavier Aldeguer

**Affiliations:** ^1^ GoodGut S.L.U, Girona, Spain; ^2^ Hospital Universitari de Bellvitge, l’Hospitalet de Llobregat, Barcelona, Spain; ^3^ Hospital Universitari de Girona Doctor Josep Trueta, Girona, Spain; ^4^ Institut d’Investigació Biomèdica de Girona—IdIBGi Girona, Girona, Spain; ^5^ Hospital Germans Tries i Pujol, CIBEREHD Badalona, Badalona, Spain; ^6^ Biology Department, University of Girona, Girona, Spain

**Keywords:** Crohn’s disease, gut microbiota, endoscopic recurrence, precision medicine, surgical resection

## Abstract

**Background and aims:** Although there are several effective drugs for the treatment of Crohn’s disease (CD), almost 70% of patients will require surgical resection during their lifetime. This procedure is not always curative, as endoscopic recurrence occurs in 65%–90% of patients in the first year after surgery. The aetiology of the recurrence is unknown; however, several studies have shown how the resident microbiota is modified after surgery. The aim of this study was to evaluate samples from patients with Crohn’s disease before and after an intestinal resection to determine whether there were differences in the abundance of different microbial markers, which may predict endoscopic recurrence at baseline.

**Methods:** In this observational study, a stool sample was obtained from 25 patients with Crohn’s disease before undergoing surgery, recruited at three Catalan hospitals. From each sample, DNA was purified and the relative abundance of nine microbial markers was quantified using qPCR.

**Results:** An algorithm composed of four microbial markers (*E. coli*, *F. prausnitzii phylogroup I*, *Bacteroidetes*, and *Eubacteria*) showed a sensitivity and specificity of 90.91% and 85.71%, respectively, and a positive and negative predictive value of 83.33% and 92.31%, respectively.

**Conclusion:** A microbial signature to determine patients who will have post-surgical recurrence was identified. This tool might be very useful in daily clinical practice, allowing the scheduling of personalized therapy and enabling preventive treatment only in patients who really require it.

## 1 Introduction

Crohn’s disease (CD) is a chronic inflammatory bowel disease (IBD) with symptoms evolving in a relapsing and remitting manner that leads to bowel damage and disability (Baumgart and Sandborn, 2012). All segments of the gastrointestinal tract may affected, most commonly the terminal ileum and colon (Torres et al., 2017). Even with the advent of immunomodulatory therapies, up to 70% of CD patients require intestinal resection during their lifetime (Hamilton et al., 2020), of whom subclinical endoscopic recurrence occurs in the anastomosis in 90% and approximately two-thirds require further surgery in the first year after surgery (De Cruz et al., 2015). Given the significant risk of recurrence after surgery, many clinical factors have been shown to predispose to recurrence, including smoking, younger age of disease onset, length of the resected segment and disease behaviour, although these factors are far from being adequate in predicting recurrence (Gklavas et al., 2017; Zhuang et al., 2021). Currently, the gold standard for diagnosing recurrence is to calculate the Rutgeerts score by performing a colonoscopy within the first year after surgery to adapt treatment (Rutgeerts. et al., 1990). However, early therapeutic intervention to prevent disease recurrence is recommended after surgery (Ding et al., 2016).

Microbiome studies have linked IBD to an alteration in gut microbiota communities in comparison with the predominant composition in healthy controls, and this is known to play a key role in disease initiation and maintenance (Manichanh et al., 2012; Manichanh, 2006; Joossens et al., 2011; Macfarlane et al., 2009; Pascal et al., 2017). Post-operative disease recurrence has also been related to the microbiota, with large differences found between patients with and without recurrence before undergoing surgery (Wright et al., 2017; Sokol et al., 2019; Hamilton et al., 2020). This suggests there may be a microbial signature, detectable before resection, that may be associated with the risk of postoperative recurrence. A surgical prognosis would permit the tailoring the treatment of patients with CD undergoing surgery, allowing early detection and treatment management and, in consequence, improving the quality of life.

Therefore, the aim of this study was to assess whether, at baseline (before surgery), there are differences in the abundance of different microbial markers depending on whether patients undergoing surgery develop endoscopic recurrence, and to evaluate the impact of ileocolonic resection on the abundance of gut microbiota species and whether endoscopic recurrence may be predicted at baseline by a single microbial marker or the definition of a microbial signature capable of differentiating between groups with high diagnostic capacity.

## 2 Materials and methods

### 2.1 Study population

The study cohort comprised 25 CD patients scheduled for ileocecal resection surgery recruited between April 2018 and July 2020. This multicentre randomized study was conducted in three centres: The Department of Gastroenterology, Hospital Universitari Dr. Josep Trueta (Girona, Catalonia), Hospital Universitari de Bellvitge (Barcelona, Catalonia), and Hospital Germans Trias i Pujol (Badalona, Catalonia). Ethical approval was obtained from all participating hospitals. Patients underwent habitual clinical procedures. Informed consent was obtained from each patient.

The inclusion criteria were age >18 years, signed informed consent, ileal or ileocolonic CD, and an indication for CD-related intestinal surgery (ileocolonic resection) for the first time. Subjects were excluded if they had received antibiotics in the month before the operation, were pregnant at inclusion or had a history of illness and/or surgery that compromised the digestive tract.

Baseline clinical characteristics of patients are shown in Table 1. A complete resection was systematically made, with all margins free from inflammation.

**TABLE 1:** Patient characteristics.

Demographics	Recurrence (Rutgeerts i0-i1)	Remission (Rutgeerts i2-i4)
N, (male)	11 (8)	14 (4)
Mean age at surgery	35	41
Age range	21–47	21–61
Age at diagnosis	N (%)	N (%)
A1 ≤ 16 Year	1 (9.1%)	—
A2 17–40	8 (72.7%)	11 (78,6%)
A3 >40	2 (18.2%)	3 (21.4%)
Disease location at surgery	N (%)	N (%)
L1 Ileum only	6 (54.5%)	9 (64.3%)
L2 Colon only	—	—
L3 Ileum and colon	5 (45.5%)	5 (35.7%)
L4 Upper GI	—	—
Disease phenotype at surgery	N (%)	N (%)
B1 Inflammatory	1 (9.1%)	1 (7.1%)
B2 Stricturing	4 (36.3%)	8 (57.2%)
B3 Penetrating	5 (45.5%)	3 (21.4%)
P Perianal disease	1 (9.1%)	2 (14.3%)

Study patients were asked to collect a faecal sample no earlier than 1 week before surgery and at 3 and 6 months post-surgery. To ensure sample viability, they had to be stored in one of the following conditions according to the time elapsed between sample deposition and its arrival at the hospital: 1) If the time elapsed was <4 h, the sample could be stored at room temperature, 2) if the time elapsed between sampling and arrival at the hospital was more than 4 but less than 12 h, it had to be kept in a refrigerator at 4 C, and 3) if the sample took more than 12 h before arrival at the hospital, it had to be frozen at −20°C. Samples were kept frozen at −20°C once received at the hospital and stored at −80°C upon analysis.

A stool sample related to colonoscopy results was collected 6 months post-surgery. These samples were asked to be collected prior to bowel preparation, and frozen immediately after deposition at −20°C. The Rutgeerts index was used to assess endoscopic recurrence at approximately 6 months post-surgery, and recurrence was defined as a Rutgeerts score ≥ i2.

### 2.2 DNA extraction from stool samples

Total DNA was isolated from faecal samples using the NucleoSpin Soil DNA kit (Macherey-Nagel GmbH &Co., Düren, Germany), according to the manufacturer’s instructions, and eluting DNA in a 100 μL final volume of SE Elution.

### 2.3 qPCR analysis

The abundance of nine microbial markers was analysed using qPCR: Eubacteria (EUB), *Faecalibacterium prausnitzii* (FAE), and its two phylogroups (PHGI, PHGII), *Akkermansia muciniphila* (AKK), *Escherichia coli* (ECO), Bacteroidetes (BAC), *Ruminococcus* spp. (RUM), and *Methanobrevibacter smithii* (MSM). The targeting primers and probes used are shown in Table 2. These microbial species or taxa were chosen as a representative of the gut microbiota: Eubacteria was analysed as the total bacterial load representation (Nadkarni et al., 2002); *Ruminococcus* spp, *F. prausnitzii* and its phylogroups were analysed as butyrate-producing bacteria and as indicators of the mucosa homeostasis together with *A. muciniphila,* which have been related to IBD (Hanauer, 2019; la Reau and Suen, 2018; Derrien et al., 2017; Miquel et al., 2013; Watterlot et al., 2008; Lopez-Siles et al., 2014a); *E. coli* was analysed as a pro-inflammatory bacteria; *M. smithii* as a methanogen representation; and Bacteroidetes as a representative of mucosal equilibrium.

**TABLE 2:** 16S rRNA-targeted primers and probes sequences used in this study. The fluorophore and the quencher of the probes are also shown. NA, not applicable.

Target microbial marker	Primers and probes			Concentration (nM)	
	Code	Sequence 5′→3′	Reference	Forward and reverse primers	Probe
*F. prausnitzii*	FAE_F	TGT​AAA​CTC​CTG​TTG​TTG​AGG​AAG​ATA​A	Lopez-Siles et al. (2014b)	250	300
	FAE_R	GCGCTCCCTTTACACCCA			
	FAE_PR	FAM-CAAGGAAGTGACGGCTAACTACGTGCCAG-TAMRA			
*F. prausnitzii* (phylogroups)	PHG_F	CTC​AAA​GAG​GGG​GAC​AAC​AGT​T	Lopez-Siles et al. (2016)	300	900
	PHG_R	GCC​ATC​TCA​AAG​CGG​ATT​G			
	PHGI_PR	FAM-TAAGCCCACGACCCGGCATCG-BHQ1			
	PHGII_PR	HEX-TAAGCCCACRGCTCGGCATC-BHQ1			
*A. muciniphila*	AKK_F	CAG​CAC​GTG​AAG​GTG​GGG​AC	Collado et al. (2007)	250	NA
	AKK_R	CCT​TGC​GGT​TGG​CTT​CAG​AT			
*Ruminococcus* spp.	RUM_F	GGCGGCYTRCTGGGCTTT	Ramirez-Farias et al. (2009)	250	NA
	RUM_R	CCAGGTGGATWACTTATTGTGTTAA			
*M. smithii*	MSM_F	ACG​CAG​CTT​AAA​CCA​CAG​TC	Ramió‐Pujol et al. (2020)	200	NA
	MSM_R	AAAGACATTGACCCRCGCAT			
*E. coli*	ECO_F	CAT​GCC​GCG​TGT​ATG​AAG​AA	Huijsdens et al. (2002)	300	100
	ECO_R	CGG​GTA​ACG​TCA​ATG​AGC​AAA			
	ECO_PR	TAT​TAA​CTT​TAC​TCC​CTT​CCT​CCC​CGC​TGA​A			
Bacteroidetes	BAC_F	CRAACAGGATTAGATACCCT	Bacchetti De Gregoris et al. (2011)	300	NA
	BAC_R	GGT​AAG​GTT​CCT​CGC​GTA​T			
Eubacteria	EUB_F	ACT​CCT​ACG​GGA​GGC​AGC​AGT	Nadkarni et al. (2002)	200	NA
	EUB_R	GTA​TTA​CCG​CGG​CTG​CTG​GCA​C			

EUB, AKK, RUM, MSM, and BAC were quantified by preparing single reactions of each biomarker using GoTaq qPCR Bryt Master mix (Promega, Madison, United States). Reactions consisted of 10 μL containing 1X GoTaq^®^ qPCR Master Mix (Promega), between 200 and 300 nM of each primer (specified in Table 2), and between 12 and 20 ng of genomic DNA template. FPRA, PHGI, PHGII, and ECO were quantified by preparing a single reaction for each biomarker using GoTaq qPCR Probe Master Mix (Promega. Madison, United States). Reactions consisted of 10 μL containing 1X GoTaq^®^ qPCR Master Mix (Promega), 300 nM of each primer, between 100 and 250 nM of each probe (specified in Table 3), and between 12 and 20 ng of genomic DNA template. The primers used were purchased from Macrogen (Macrogen, Seoul, South Korea).

**TABLE 3:** Sensitivity, specificity, and area under the curve (AUC) for faecal calprotectin and relative abundance of Bacteroidetes (BAC), *A. muciniphila* (AKK), *Ruminococcus* spp. (RUM), *E. coli* (ECO), *F. prausnitzii* (FAE) and its two pyhlogroups (PHGI and PHGII), and *M. smithii* (MSM).

Marker	Sensitivity (%)	Specificity (%)	AUC
Faecal calprotectin	100	62.5	0.875
BAC	100	36.4	0.737
AKK	66.7	54.5	0.444
RUM	77.8	44.5	0.586
ECO	77.8	36.4	0.576
FAE	88.9	54.5	0.596
PHGI	66.7	36.4	0.576
PHGII	88.9	54.5	0.626
MSM	66.7	36.4	0.384

All quantitative PCRs were run on an AriaMx Real-time PCR System (Agilent Technologies, Santa Clara, United States). Thermal profiles of all qPCRs included an initial denaturation step set at 95°C for 10 min and 40 cycles with 15 s of denaturing at 95°C and 1 min of elongation at 60°C (except for FPRA phylogroups, in which the elongation step was at 64°C). A melting curve step was added at the end of each qPCR with GoTaq qPCR Bryt Master Mix and was used to verify the presence of the expected amplicon size and to control primer dimer formation when applicable. All samples were amplified in duplicate, which were considered valid when the difference between threshold cycles (Ct) was <0.6. A non-template control and a positive control reaction were included in each qPCR run.

Quantification data of each microbial marker was collected and analysed using Aria Software version 1.71 (Agilent Technologies, Santa Clara, United States).

Once the results were obtained, data were normalised by Eubacteria to avoid differences due to laboratory procedures or the abundance of biomarkers.

### 2.4 Calprotectin quantification

Calprotectin is a calcium-binding protein expressed in neutrophils and monocytes and is used as an inflammatory marker for gastrointestinal diseases (Stříž and Trebichavský, 2004; Ayling and Kok, 2018). Elevated levels of calprotectin in faeces are an indicator of active disease, making it a widely used marker to monitor therapy in patients with IBD (Boschetti et al., 2015). The inflammatory state was quantified by measuring the amount of faecal calprotectin using the PhicalR ELISA Immunodiagnostic Test. The analysis was made at LABCO (Barcelona, Spain). Levels >250 μg/g were associated with remission.

### 2.5 Statistical analysis

Data normality was assessed using the Kolmogorov-Smirnov test. The non-parametric Kruskal–Wallis test was used to test differences in variables with more than two categories. Pairwise comparisons of subcategories of these variables were analysed using the non-parametric Mann-Whitney test. Paired data were analysed using the McNemar test and correlations using the Pearson test.

The receiver operating characteristic (ROC) curve was used to determine the usefulness of each biomarker in distinguishing between patients with and without post-surgical recurrence. The accuracy of discrimination was measured by the area under the ROC curve (AUC). An AUC approaching 1 indicates that the test is highly sensitive and specific whereas an AUC approaching 0.5 indicates that the test is neither sensitive nor specific. Chi-square tests were performed to assess the influence of categorical variables on the response variable.

All results using microbial markers were performed using their relative abundances (Ct value of each biomarker normalized by EUB Ct value). This normalized the results and the effects derived from the process such as the DNA concentration of each sample were eliminated.

Sensitivity, specificity, and positive and negative predictive values of the algorithm designed were calculated using Epidat 3.1 (SERGAS, Xunta de Galicia, Spain). All remaining statistical analyses were performed using the SPSS 23.0 statistical package (IBM, NYC, United States). Significance levels were established as *p* = ≤ 0.05.

The final algorithm is based on the decision abundance (DA) calculated using the following equation:

$$DA=\frac{\frac{{Ct}_{ind}-b_{ind}}{m_{ind}}}{\frac{{Ct}_{EUB}-b_{EUB}}{m_{EUB}}}$$ (1)

Where CT is the threshold cycle; b is the intercept point; m is the slope; ind, is the microbial marker; and EUB is eubacteria (total bacterial load).

## 3 Results

### 3.1 Microbial marker profile

**FIGURE 1:**
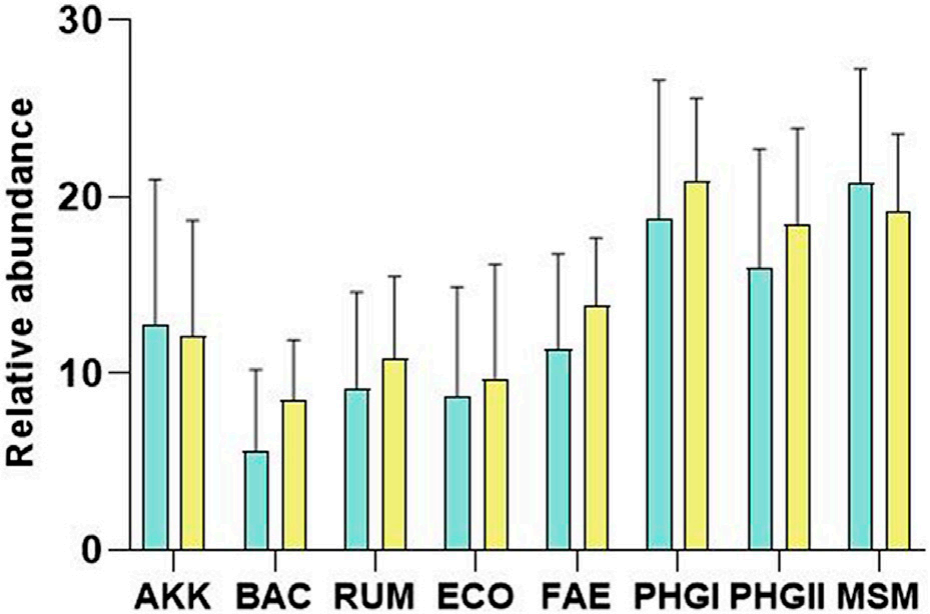
Plot of the relative abundances (Microbial marker Ct—Eubacteria Ct) of *A. muciniphila* (AKK), Bacteroidetes (BAC), *Ruminococcus* spp. (RUM), *E. coli* (ECO), *F. prausnitzii* (FAE), *F. prausnitzii* phylogroup I (PHGI), *F. prausnitzii* phylogroup II (PHGII), *M. smithii* (MSM) for remission (green) and recurrence patients (yellow). No significant differences were found (*p*-value>0.05). Note that results are shown in PCR Ct values, so a higher value indicates lower abundance than a lower value.

Sample A (1 week before surgery) was used to analyse whether microbial markers could predict whether there would be post-surgical recurrence. No significant differences were observed between microbial markers when the two groups (recurrence and no recurrence) were compared (*p*-value >0.05) (Figure 1).

Table 3 shows the accuracy of discrimination measured using the area under the ROC curve (AUC) for all microbial markers, with the best sensitivity and specificity results. Calprotectin was the biomarker with the greatest discriminatory capacity and the highest specificity when the remission and the recurrence groups were compared. To prevent DNA quantity bias, relative abundances were calculated by subtracting the value of the total bacterial load (Eubacteria) from the value of each specific marker. The relative abundance of BAC had the highest AUC of microbial markers, with the same sensitivity as calprotectin but less specificity (36.4%). Despite this, the microbial markers were not sufficient to individually discriminate between the two groups of patients.

### 3.2 Combination of microbial markers to predict post-surgical recurrence

Initially, the specific patient factors were analysed to measure their influence on remission or recurrence. Chi-square tests showed that these factors did not influence the response to our data, with a *p*-value of 0.916 for onset age, 0.821 for disease location, 0.149 for disease phenotype, and 0.087 for patient gender.

Since a single marker could not discriminate between the two target groups with high sensitivity and specificity, we combined different microbial markers by designing a mathematical algorithm. The algorithm defined had the best predictive capacity to define the surgical prognosis.

Specifically, it combines the relative abundance of three microbial markers (PHGI-EUB, BAC-EUB, and ECO-EUB) where lower counts of PHGI and BAC and higher counts of ECO were associated with recurrence. The algorithm showed a high capacity to discriminate between patients with and without post-surgical recurrence, with a sensitivity and specificity of 90.91% and 85.71%, respectively, and a positive and negative predictive value of 83.33% and 92.31%, respectively (Table 4).

**TABLE 4:** Sensitivity, specificity, positive predictive value, and negative predictive value obtained with the algorithm.

Remission vs. Recurrence	Sensitivity (%)
Sensitivity (%)	90.91
Specificity (%)	85.71
Positive predictive value	83.33
Negative predictive value	92.31

### 3.3 Evolution of abundance of microbial markers after surgery

The abundance of the microbial markers analysed in stool samples collected 3 and 6 months after surgery were compared with those collected pre-surgery. Patients with remission or relapse post-surgery were analysed separately.

**FIGURE 2:**
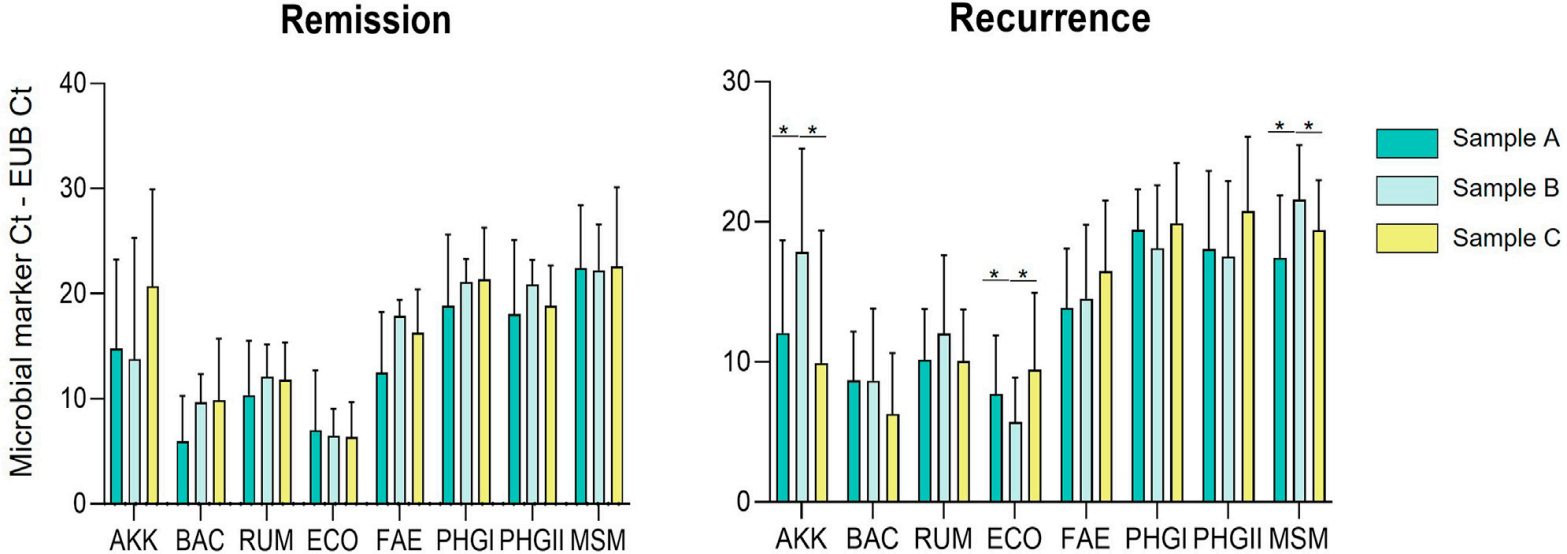
Plot of the relative abundances (Microbial marker Ct—Eubacteria Ct) of *A. muciniphila* (AKK), Bacteroidetes (BAC), *Ruminococcus* spp. (RUM), *E. coli* (ECO), *F. prausnitzii* (FAE), *F. prausnitzii* phylogroup I (PHGI), *F. prausnitzii* phylogroup II (PHGII), *M. smithii* (MSM) for sample A (before surgery), sample B (3 months after surgery) and sample C (6 months after surgery). Note that results are shown in PCR Ct values, so a higher value indicates lower abundance than a lower value.

Significant differences were only observed in the postoperative recurrence group, whereas in the remission group the abundance of the markers remained stable over time (Figure 2).

The markers that presented changes in abundance post-surgery were AKK, MSM and ECO; the first two showed a decrease in abundance post-surgery and there was an increase in ECO abundance (all *p*-value = 0.028). As shown in Figure 2, this variation was observed when comparing sample A (pre-surgery) with sample B (3 months later). However, in sample C (6 months post-surgery) the abundance of the markers that showed changes reverted to the levels observed pre-surgery.

**FIGURE 3:**
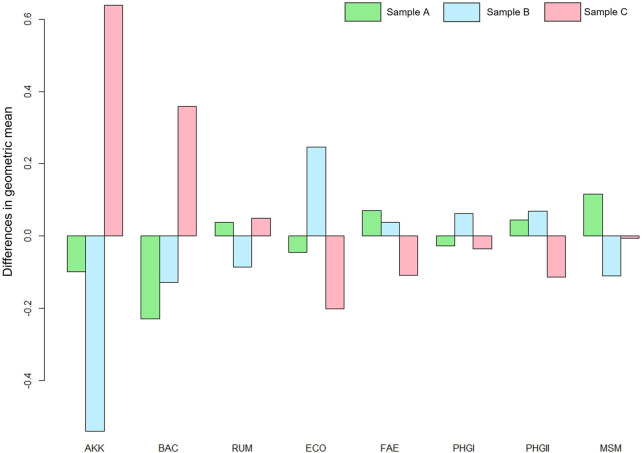
MANOVA plot where the effects of the surgery on the markers are observed. *A. muciniphila* (AKK), Bacteroidetes (BAC), *Ruminococcus* spp. (RUM), *E. coli* (ECO), *F. prausnitzii* (FAE), *F. prausnitzii* phylogroup I (PHGI), *F. prausnitzii* phylogroup II (PHGII), *M. smithii* (MSM) and for sample A (before surgery), sample B (3 months after surgery) and sample C (6 months after surgery).

Figure 3 shows the effects of surgery on the different markers of patients with post-surgical recurrence in more detail, emphasizing the significant differences observed in the AKK, MSM, and ECO markers from the MANOVA analysis performed.

## 4 Discussion

Endoscopic recurrence can develop a few days post-surgery and is dependent on the return of the faecal stream and re-establishment of faecal microbiota (Haens et al., 1998). Numerous studies have shown that alterations in the gut microbial profile at the time of surgery or the post-operative follow-up are linked to the post-operative disease course in CD patients (Sokol et al., 2019; Hamilton et al., 2020; Strömbeck et al., 2020; Zhuang et al., 2021). Our results show that, from baseline, there are some differences between the microbial communities of patients with and without relapse. Although these differences were observed in only three of the markers analysed, which may be insufficient to show differences in the microbial ecosystem between groups, we believe that it is sufficient to suggest that patients who relapse have a more unstable and delicate microbiota, since resection affects them more.

The combination of three different species as a predictive tool for post-operative recurrence provides more robust and reliable results. The proposed algorithm combines the relative abundance of three microbial markers (ECO/EUB, PHGI/EUB, and BAC/EUB).

An increase in the abundance *of E. coli* has been associated with the early recurrence of CD in several studies (Neut et al., 2002; Doherty et al., 2010). As expected, in our study, the counts of *E. coli* in faeces after surgery increased in patients with recurrence. Similarly, whereas we did not observe differences in the abundance of *F. prausnitzii* or its two phylogroups, lower counts of *F. prausnitzii* phylogroup I were related to recurrence in the algorithm designed. *F. prausnitzii* is one of the most abundant commensal butyrate-producing bacteria in the microbiota of healthy subjects, and its prevalence and abundance has been shown to be reduced in IBD patients (Sokol et al., 2008; Miquel et al., 2013; Lopez-Siles et al., 2014b). Butyrate is a short-chain fatty acid (SCFA), which are volatile fatty acids produced by the gut microbiota in the large bowel as a fermentation product from undigested components. The most abundant SCFA are acetic, propionic, and butyric acid (Ríos-Covián et al., 2016). Our study highlights that, among the benefits of butyrate, a strong effect is produced in IBD, probably as a consequence of mucosal healing and inhibition of inflammation (Russo et al., 2019). This confirms that the clearance abundance of these SCFA-producing bacteria is correlated with a greater predisposition to relapse. Moreover, other studies have found that a low level of SCFA-producing bacteria may be associated with postoperative endoscopic recurrence (Mondot et al., 2016; Wright et al., 2017; Sokol et al., 2019).

In addition, a depleted abundance of Bacteroidetes phylum was paired with endoscopic recurrence in the algorithm designed, which fits with its contribution to gut health. Other studies have suggested that Bacteroidetes species contribute to the homeostasis of the gut microbiota, synthetizing compounds with immunomodulatory properties such as linoleic acid, and that, by improving its diversity and composition, the wellbeing of IBD patients is enhanced (Lee et al., 2013; Delday et al., 2019; Nomura et al., 2021).

The algorithm defined has 90.91% sensitivity and 85.71% specificity, which could lead to the identification of patients prone to recurrence post-surgery, permitting treatment to be personalised, which would increase patients’ quality of life and have benefits related to healthcare costs. This would be an improvement in IBD management since there is currently no test that allows the effectiveness of intestinal resection to be predicted.

With respect to the results observed on the effect of surgery on the intestinal microbiota, we found that the counts of the acetate short-chain fatty acid (SCFA)-producing bacteria such as *A. muciniphila* were reduced. Acetate is the most prominent SCFA and substrate for butyrate production and is reported to have effects on lipid metabolism, such as lipogenesis and cholesterogenesis (Morrison et al., 2016). Similarly, the abundance of *Methanobrevibacter smithii* was reduced 3 months post-surgery in patients prone to recurrence, which was reverted at 6 months. *Methanobrevibacter smithii* is an archaeon that converts CO_2_ and H_2_ into CH_4_ and also produces SCFA from carbohydrates; its prevalence in stool samples from IBD patients is significantly lower than in healthy control subjects (Bang et al., 2014). In addition, methane levels are lower in IBD patients than in healthy subjects (Chaudhary et al., 2018).

Our results show intestinal surgery changes the abundance of some of the microbial markers in patients with post-surgical recurrence, but the changes were reverted at 6 months. In contrast, in patients who go into remission, the abundance of no marker was altered, demonstrating a much more stable microbiota from baseline.

The study had some limitations. Despite the promising results obtained, the study was performed in a small cohort and more robust examination in further large-scale studies is needed to validate the algorithm defined prior to its application in clinical practice. Secondly, all the results are based on faecal microbiota and neither data from ileal mucosa-associated microbiota was available nor was a metagenomic analysis performed. However, our markers were initially determined from intestinal biopsies from mucosa. The quantification of these markers was further optimized in stool samples, avoiding one of the drawbacks of clinical microbiology applied to stool markers, which is their vulnerability to dietary changes in different populations (Lopez-Siles et al., 2017). Thirdly, clinical risk factors such as smoking and the surgical margins inflammation, both important in predicting recurrence were not analysed. Despite this, the objective of our study was to define an algorithm that worked independently of these factors facilitating its use. All these limitations could be addressed with a larger prospective study, which we plan to carry out in the future.

In conclusion, this study shows that surgery has an impact on the microbiota and that postoperative endoscopic recurrence is associated with changes in its composition and abundance. Precision medicine and biomarker-guided surgery prognosis are a necessary step in advancing the clinical effectiveness and improving the quality of life. Hence, the gut microbiota has the potential to become a precise tool for predicting the postoperative evolution and recurrence, which would allow, through microbial therapies, redirection of the microbial profile, and the avoidance of postoperative recurrence.

## Data Availability

The raw data supporting the conclusion of this article will be made available by the authors, without undue reservation.
